# Trace gluten contamination may play a role in mucosal and clinical recovery in a subgroup of diet-adherent non-responsive celiac disease patients

**DOI:** 10.1186/1471-230X-13-40

**Published:** 2013-02-28

**Authors:** Justin R Hollon, Pamela A Cureton, Margaret L Martin, Elaine L Leonard Puppa, Alessio Fasano

**Affiliations:** 1 Division of Pediatric Gastroenterology and Nutrition, Johns Hopkins University School of Medicine, 600 North Wolfe Street, Brady 320, Baltimore, MD, USA; 2 University of Maryland School of Medicine, Baltimore, MD, USA; 3 Center for Celiac Research, Massachusetts General Hospital and Division of Pediatric Gastroenterology and Nutrition, Massachusetts General Hospital for Children, Boston, MA, USA

**Keywords:** Celiac disease, Refractory celiac disease, Refractory sprue, Non-responsive celiac disease, Gluten-free diet

## Abstract

**Background:**

Patients with persistent symptoms and/or villous atrophy despite strict adherence to a gluten-free diet (GFD) have non-responsive celiac disease (NRCD). A subset of these patients has refractory celiac disease (RCD), yet some NRCD patients may simply be reacting to gluten cross-contamination. Here we describe the effects of a 3-6 month diet of whole, unprocessed foods, termed the Gluten Contamination Elimination Diet (GCED), on NRCD. We aim to demonstrate that this diet reclassifies the majority of patients thought to have RCD type 1 (RCD1).

**Methods:**

We reviewed the records of all GFD-adherent NRCD patients cared for in our celiac center from 2005-2011 who were documented to have started the GCED. Response to the GCED was defined as being asymptomatic after the diet, with normal villous architecture on repeat biopsy, if performed.

**Results:**

Prior to the GCED, all patients were interviewed by an experienced dietitian and no sources of hidden gluten ingestion were identified. 17 patients completed the GCED; 15 were female (88%). Median age at start of the GCED was 42 years (range 6-73). Fourteen patients (82%) responded to the GCED. Six patients met criteria for RCD prior to the GCED; 5 (83%) were asymptomatic after the GCED and no longer meet RCD criteria. Of the 14 patients who responded to the GCED, 11 (79%) successfully returned to a traditional GFD without resurgence of symptoms.

**Conclusions:**

The GCED may be an effective therapeutic option for GFD-adherent NRCD patients. Response to this diet identifies a subgroup of patients, previously classified as RCD1, that is not truly refractory to dietary treatment. Preventing an inaccurate diagnosis of RCD1 avoids immunotherapy. Most patients are able to return to a traditional GFD without return of symptoms.

## Background

Celiac disease (CD) is an immune-mediated small intestinal enteropathy triggered by the ingestion of gluten in the genetically susceptible, with prevalence in the United States of nearly 1% [1]. While this inflammatory disease manifests classically with GI symptoms, including diarrhea, malabsorption and weight loss, more commonly patients are asymptomatic or present with extraintestinal manifestations to include iron-deficiency anemia, osteoporosis, migraines, depression and autoimmune disease comorbidities [2,3]. The cornerstone of treatment for CD is the gluten free diet (GFD), with the vast majority of patients demonstrating substantial clinical improvement within the first few weeks to months after diet initiation [4,5]. However, there is a subset of patients who have so-called non-responsive celiac disease (NRCD) in that they either fail to ever respond to the GFD or have a recurrence/relapse of symptoms despite being on a GFD [6].

By far the most common cause of non-response is failure to adhere to the prescribed GFD, either voluntarily or unintentionally, thus highlighting the importance of a comprehensive dietary assessment [7,8]. Additionally, persistently symptomatic patients may be suffering from co-morbidities such as inflammatory bowel disease or microscopic colitis rather than active celiac disease [6]. However, a portion of the symptomatic NRCD patient population are truly on a strict GFD and, of particular concern, those with villous atrophy on repeat small intestinal biopsy meet the definition of refractory sprue/refractory celiac disease (RCD) [6,9-11]. Based on the immunophenotype of the intraepithelial lymphocytes (IEL) population, RCD may be further divided into type 1 and type 2; RCD1 demonstrates a normal polyclonal IEL population, whereas RCD2 is defined by the presence of aberrant monoclonal IELs [9,12]. Although RCD1 patients are at increased risk of celiac-associated complications such as growth retardation and osteoporosis [13], prognostically, RCD 1 follows a much more benign and indolent course than RCD2. In contrast to the 5 year survival rate approaching 100% in RCD1, the more aggressive RCD2 carries a higher risk of developing enteropathy-associated T-cell lymphoma (ETCL), and overall leads to a shortened life expectancy [9,10,14-17], with a 5 year mortality estimated between 50-60% [14,16]. Corticosteroids are typically first-line therapy for both RCD1 and RCD2, yet the risk remains that a subset of RCD1 patients, despite strictly adhering to a proper GFD, are simply reacting to trace amounts of gluten; below the 20 parts per million (ppm) threshold considered safe for the vast majority of CD patients.

The University of Maryland Center for Celiac Research in Baltimore, MD is a tertiary referral center for celiac disease and gluten-related disorders. As a celiac specialty clinic, many persistently symptomatic celiac patients are referred to our center for further evaluation and management of presumed RCD. Our initial evaluation of an NRCD patient begins with a comprehensive assessment of adherence to the diet by an expert dietitian, with focus not only on intentional noncompliance but also on determining the patient’s awareness regarding hidden sources of gluten to include communion wafers, medications and additives [9,18,19]. Whereas treatment for the non-adherent patient is relatively straight-forward, difficulty arises when faced with the NRCD patient who has evidence of persistent disease but no identifiable source of continued gluten ingestion. Typically, such patients have already sought out second/third opinions, have had their diet scrutinized by multiple dietitians and may have initiated steroid treatment for presumed RCD. Nevertheless, despite following a strict GFD, there is a possibility that these patients are still being exposed to, and are sensitive to, a degree of gluten typically tolerated by the vast majority of CD patients. In 2005, as a first step for the management of such NRCD cases, we began to prescribe a modified diet that aims to eliminate any possible sources of gluten cross-contamination in an already strict GFD. If successful, this diet, referred to as the Gluten Contamination Elimination Diet (GCED), would not only result in symptom resolution, but would avoid the inaccurate diagnosis of RCD and, thus, avoid the expense and adverse health effects of steroid therapy or other immunomodulators. The aim of this single-center retrospective chart review is to evaluate and present our 7-year experience of using the GCED to treat NRCD patients that have failed a well-documented strict GFD.

## Methods

This study was approved by the Institutional Review Board of the University of Maryland, Baltimore (IRB), and was conducted in accordance with the ethical standards and regulatory requirements of the IRB. We define NRCD as biopsy-proven CD with persistence or relapse of symptoms *and/or* villous atrophy despite being on a gluten free diet for ≥12 months. Prior to being considered for the GCED, dietary compliance was assessed by an experienced dietitian with expertise in celiac disease; patients must have been strictly adherent to the GFD, with no identifiable source of continued gluten exposure. Histological changes on duodenal biopsies were staged according to the Marsh Oberhuber classification [20]. RCD is defined as persistent or relapse of symptoms *and* villous atrophy (Marsh 3), despite a strict GFD for ≥12 months. If IEL immunophenotyping was performed on biopsy specimens, either by CD3/CD8 immunostaining or by T-cell receptor gene rearrangement analysis by polymerase chain reaction (PCR), RCD is further subdivided into RCD1 or RCD2 depending on the absence or presence, respectively, of a monoclonal IEL population. Celiac serology (IgA antibodies to tissue transglutaminase (tTG) and/or anti-endomysial antibodies (EMA)) is defined as negative, weak positive or high using the cut-offs provided by the ELISA kit manufacturer. Response to the GCED is defined as being asymptomatic after the diet, with normal villous architecture (Marsh 0-2) on repeat biopsy, if performed. Although celiac serology is drawn prior to initiation of the GCED and upon follow-up, the presence or absence of celiac auto-antibodies is not used in the definition for NRCD or RCD, nor is normalization of celiac serology, if applicable, used as a criterion for response to the GCED.

The GCED, as shown in Table 1, allows: brown and white rice; all fresh (no frozen, canned or dried) fruits, vegetables and herbs; fresh meats, poultry, fish and other non-processed protein sources. Unflavored, unseasoned dairy products are introduced on week 4. Allowed condiments are oils, vinegar (excluding flavored and malt vinegars), honey and salt. Allowed beverages are 100% fruit/vegetable juices, Gatorade, milk, water, and gluten-free supplemental formulas such as Boost and Ensure. All cereal grains aside from rice are prohibited. Processed cheeses, lunch meats, ham, bacon or other such processed, self-basted or cured meat products are not allowed. Lastly, a gluten-free multivitamin/mineral daily supplement is recommended and prescription medication (verified as gluten-free) is continued. Patients are provided with sample menus and are asked to keep a food record. As an aid for compliance, contact information for the dietitian is provided and patients are encouraged to update the dietitian frequently on their progress and to ask questions as they arise. They are instructed to return to clinic in 3 months for repeat celiac serology labwork and symptom re-evaluation.

**Table 1:** Products allowed/disallowed in the Gluten Contamination Elimination Diet (GCED), targeting the elimination of gluten cross-contamination

	**Allowed**	**Not allowed**
Grains	Plain, unflavored, brown and white rice	Millet, sorghum, buckwheat or other inherently gluten-free grains, seeds, or flours
Fruits/Vegetables	All fresh fruits/vegetables	Frozen, canned or dried
Proteins	Fresh meats	Lunch meats
	Fresh fish	Ham, bacon
	Eggs	Other processed, self-basted or cured meat products
	Dried beans	
	Unseasoned nuts in the shell	
Dairy	Butter, yogurt (unflavored), milk (unflavored), aged cheeses	Seasoned or flavored dairy products
		Processed cheeses
Condiments	Oils, vinegar, honey, salt	Flavored and malt vinegars
Beverages	100% fruit/vegetable	
	Gluten-free supplemental formulas	
	Gatorade, milk, water	

We reviewed the charts of all patients cared for in our center from 2005 through 2011 who were instructed on the GCED and met above criteria for NRCD patients on a well-documented strict GFD. The list of patients receiving dietary instruction was drawn from the celiac dietitian database. Patients who did not follow-up in clinic or otherwise communicate with our clinic after diet education were excluded as data were unavailable, to include whether or not the diet was initiated. Compliance was determined by patient report and dietitian interview; declaration of stopping the diet before the prescribed minimum of 3 months is considered noncompliance.

## Results

Of the 1,288 patients with CD seen by our center from 2005 to 2011, a total of 29 (2.3%) patients were instructed on the GCED and met criteria for diet-adherent NRCD. Eight of these had no further communication with our clinic (lost to follow-up). The remaining 21 patients were documented to have started the GCED, with 4 of them admitting to cessation of the GCED and return to a traditional GFD prior to 3 months (noncompliant). Of the 17 compliant patients, 15 were female (88%). The median age of this group at initial diagnosis of CD was 30 years (range 1.6 - 52). Median age at initiation of the GCED was 42 years (range 6 – 73); 5 patients were <21 years of age. 14 (82%) patients had primary NRCD, with no improvement since starting the GFD, on average, 3 years prior to the GCED (range 1.1 - 4.6). 3 (18%) had secondary NRCD, with previous remission of disease but subsequent relapse, on average, 2 years prior to the GCED (range 1 – 3). Prior to starting the GCED, all but one patient (94%) were symptomatic, with the asymptomatic patient initiating the GCED due to high serology and persistent villous atrophy. Of the symptomatic, the most common symptoms were diarrhea (50%), followed by fatigue (31%) and abdominal pain (31%). The demographic details and clinical symptoms are shown in Table 2.

**Table 2:** Demographics and distribution of symptoms of patients completing ≥3 months of Gluten Contamination Elimination Diet (GCED)

**Variables**	**n (%)**
Female	15 (88%)
Pediatric ( <21)	5 (29%)
Median age at diagnosis of CD (range)	30 years (1.6 - 52)
Median age at start of GCED (range)	42 years (6 – 73)
Primary non-responsive	14 (82%)
Mean years since start of GFD (range)	3 years (1.1 - 4.6)
Secondary non-responsive/relapsed	3 (18%)
Mean years since relapse, while on GFD (range)	2 years (1 - 3)
Asymptomatic	1 (6%)
Symptomatic	16 (94%)
Diarrhea	8 (50%)
Fatigue	5 (31%)
Abdominal pain	5 (31%)
Bloating	3 (19%)
Constipation	2 (12.5%)
Inadequate weight gain	2 (12.5%)
Weight loss	1 (6.2%)
Persistent hypertransaminasemia	1 (6.2%)
Anxiety	1 (6.2%)
Multiple symptoms	9 (56.2%)

CD, celiac disease; GFD, gluten-free diet.

Response rate of the GCED was 82%, with 14 of the 17 compliant patients responding to the diet. Results of the GCED in terms of symptom resolution, histology and celiac serology are discussed below. Table 3 presents these categories, per patient, pre and post-GCED. Table 4 presents a summary comparison.

**Table 3:** Effect of Gluten Contamination Elimination Diet on celiac serology, main clinical symptoms and histologic findings

**Patient**	**Serology before**^**1**^	**Serology after**^**1**^	**Symptoms before**	**Symptoms after**	**Biopsy before**	**Biopsy after**
1	High	Neg	Abdominal pain	None		
2	High	Neg	Inadequate weight gain	None		
3	High	Neg	Diarrhea, fatigue	None	Marsh 3a (RCD)	Refused
4	High	Neg	Constipation	None	Marsh 3 (RCD)	
5	High	Weak Pos	None	None	Marsh 3a	Marsh 1
6	High	Weak Pos	Elevated transaminases	None	Marsh 2	
7	High	Weak Pos	Constipation, fatigue	None		Marsh 1
8	High	Weak Pos	Diarrhea	None	Marsh 1	
9	High	High	Diarrhea, abdominal pain, bloating	None	Marsh 3a (RCD)	Marsh 1
10	High	High	Fatigue, anxiety	Fatigue, anxiety		Marsh 3a (RCD1)
11	Weak Pos	Neg	Diarrhea, abdominal pain	None	Marsh 2	Marsh 0
12	Weak Pos	Neg	Diarrhea	None	Marsh 1	
13	Weak Pos	Weak Pos	Inadequate weight gain	Inadequate weight gain		
14	Neg	Neg	Diarrhea, bloating, fatigue	None	Marsh 0	
15	Neg	Neg	Abdominal pain, fatigue	None	Marsh 3a (RCD), VCE: Atrophy	VCE : normal
16	Neg	Neg	Diarrhea, weight loss	None	Marsh 3b (RCD)	Lost to follow-up
17	Neg	Neg	Diarrhea, abdominal pain, bloating	Diarrhea, abdominal pain, bloating	Marsh 3a (RCD)	VCE : atrophy

^1^ Celiac serology defined as negative, weak positive or high using the cut-offs provided by the ELISA kit manufacturer. Tissue transglutaminase (tTG) IgA was followed in 16/17 patients; anti-endomysial (EMA) IgA was followed in the remaining patient.

Pos, positive; RCD, refractory celiac disease; RCD1, refractory celiac disease type 1; VCE, video capsule endoscopy.

**Table 4:** Presence of symptoms, classification of celiac serology and Marsh grading of biopsy histology before and after treatment with Gluten Contamination Elimination Diet

**Symptoms**	**Pre-Diet: n (%)**	**Post-Diet: n (%)**
Asymptomatic	1 (6%)	14 (82%)
Symptomatic	16 (94%)	3 (18%)
**Histology**	**Pre-Diet: n (%)**	**Post-Diet: n (%)**
Biopsy obtained	12 (71%)	5 (29%)
Marsh 3	7	1
Marsh 2	2	
Marsh 0-1	3	4
**Celiac Serology**^**1**^	**Pre-Diet: n (%)**	**Post-Diet: n (%)**
High celiac serology	10 (59%)	2 (12%)
Weak positive celiac serology	3 (18%)	5 (30%)
Negative celiac serology	4 (24%)	10 (59%)

^1^ Celiac serology defined as negative, weak positive or high using the cut-offs provided by the ELISA kit manufacturer. Tissue transglutaminase (tTG) IgA was followed in 16/17 patients; anti-endomysial (EMA) IgA was followed in the remaining patient.

### Symptom resolution

Prior to starting the GCED, 16 of the 17 compliant patients (94%) were symptomatic and all but 3 patients became asymptomatic during the GCED, giving a symptom response rate of 81% (13/16). Six patients met the criteria for RCD prior to initiation of the GCED, with persistent symptoms and Marsh 3 histology. Five out of these 6 (83%) had full resolution of their symptoms after the GCED and no longer meet criteria for RCD.

### Histology

All patients had villous atrophy (Marsh 3) on histology at the time of their initial CD diagnosis. Twelve of the 17 compliant patients (71%) had repeat duodenal biopsies taken before initiation of the GCED. Seven patients demonstrated Marsh 3a/b histology 2.5 months, on average, prior to the GCED (range 0 – 5). Of these, patients 5 and 9 demonstrated complete mucosal healing on repeat endoscopy 5-6 months after starting the GCED; patient 15 had a normal follow-up video capsule endoscopy. Patient 17 did not respond symptomatically; follow-up video capsule endoscopy demonstrated villous atrophy and the patient was started on corticosteroids for RCD. Two patients demonstrated Marsh 2 histology <1 month prior to the GCED; patient 11 was re-biopsied 1 month after the GCED and demonstrated normal (Marsh 0) histology. Three patients demonstrated Marsh 0–1 histology prior to the GCED. There were two patients without pre-GCED biopsies that underwent endoscopy after completion of the GCED; patient 7 had responded to the diet symptomatically and demonstrated histologic remission (Marsh 1); patient 10 did not respond symptomatically to the diet and had persistent villous atrophy on histology, with IEL polyclonality, prompting corticosteroid treatment for RCD1.

### Celiac serology

All patients had elevated tTG celiac serology at ini-tial histologically-proven CD diagnosis. All compliant patients had celiac serology drawn prior to the GCED (average <2 months prior) and after initiation of the GCED (average <6 months). In all but one patient, tTG IgA was followed; EMA IgA was followed in a patient with type 1 diabetes since false positive tTG titers have been reported in subjects affected by type 1 diabetes [21]. Thirteen (76%) patients had positive celiac serology prior to starting the GCED; 10 patients (59%) were classified as high and the remaining 3 patients (18%) were in the weak positive range. Of the 10 patients with high serology, 8 (80%) converted to either negative serology (40%) or weak positive serology (40%) after the GCED. This differentiation between negative or weak positive conversion appeared to make no clinical or histological difference in outcome; all 8 patients responded to the GCED. Of the two patients who did not show improvement in serology, patient 9 had full symptom resolution and, on endoscopic reassessment, demonstrated complete mucosal healing. As such, despite the persistent elevated tTG IgA, this patient responded to the GCED as well, giving a response rate within the high serology category of 90%. Two of the 3 weak positive serology patients responded to the GCED and normalized their serology, and 3 of the 4 negative serology patients responded to the GCED, giving a response rate in these categories of 67% and 75% respectively.

### Return to traditional GFD

Of the 14 patients who responded to the diet, 11 (79%) successfully returned to their previous traditional GFD without resurgence of symptoms or elevated serology. The mean duration of the GCED among this group was 4.3 months (range 3 – 6 months) and mean duration of documented follow-up after initiation of the GCED was 20 months (range 1 – 62 months). Of the remaining 3 patients, patient 3 has had recurrence of his symptoms (diarrhea, fatigue) after attempting to return to a traditional GFD on at least 3 separate occasions and has elected to stay on the GCED indefinitely. Patient 6 is currently back on the GCED due to a recurrence of hypertransaminasemia and high celiac serology after returning to the traditional GFD. Patient 16 has been lost to follow-up and it is unknown whether or not the patient was able to successfully transition to a normal diet.

### Noncompliant patients

Four patients had documented initiation of the GCED but admitted to stopping the diet prior to the initial 3 month prescription. Three (75%) of these patients started the GCED solely for symptoms; they were serology negative and previously demonstrated Marsh 0-1 histology on biopsy. One patient stopped the diet after 1.5 months due to complete resolution of diarrhea and bloating, with no resurgence of symptoms at 7-month follow-up. The other 2 patients stopped the diet 1.5-2.25 months after initiation due to no improvement in their symptoms (diarrhea in one, fatigue/depression in the other). Normal-appearing mucosa was re-confirmed in both patients, one by push endoscopy (Marsh 0) and the other by video capsule endoscopy (normal). The last patient was started on the GCED for high serology, symptoms of inadequate weight gain and Marsh 3b histology 1 month prior to initiation of the diet. The patient discontinued the diet after 3 weeks, feeling it was too restrictive, and continues to have symptoms and elevated serology.

## Discussion

Our study describes the effects of a 3- 6 month diet of exclusively whole, unprocessed foods on clinical symptoms, mucosal pathology and celiac serology in patients with NRCD that were already properly following a strict GFD, as assessed by experienced dietitians with expertise in celiac disease. With the belief that the majority of such patients are continuing to react to miniscule amounts of gluten cross-contamination, the goal of this diet is to eliminate exposure to any possible source of gluten cross-contamination from packaged/processed foods, to include those labeled gluten-free. In this study, 82% of our study patients responded to the GCED, with resolution of symptoms and, when applicable, resolution of their prior celiac enteropathy. Most importantly, of the 6 study patients who met criteria for RCD prior to initiating the GCED, 5 (83%) responded to the diet; they did not, indeed, have RCD.

The GCED is not an attempt to enforce dietary adherence on a patient with detected persistent gluten ingestion, rather it modifies an already strict GFD to remove the possibility of trace gluten cross-contamination. Most CD patients can safely tolerate approximately 10 mg gluten cross-contamination daily, or 500 grams of food containing 20 ppm of gluten. However, there is a tremendous degree of variability within this population and some patients may have worsening histological changes with very low daily gluten exposure [22-24]. The only grains permitted in the GCED are brown and white rice as even inherently gluten-free cereal grains have been found to be significantly cross-contaminated with gluten, presumably via comingling at harvest, transport and/or milling/processing. For example, in a recent study that tested 22 single-ingredient inherently gluten-free grains, seeds and flours, 32% of these products contained >20 ppm gluten; one product contained 2,925 ppm of gluten [25]. Such a degree of cross-contamination illustrates how a significant amount of gluten may be ingested despite no apparent dietary indiscretions. Interestingly, the fact that the majority of the patients in our study were subsequently able to return to their previous strict GFD suggests that there is a degree of recovery that, once established, shifts these patients back to a more typical threshold of gluten reactivity.

**Figure 1:**
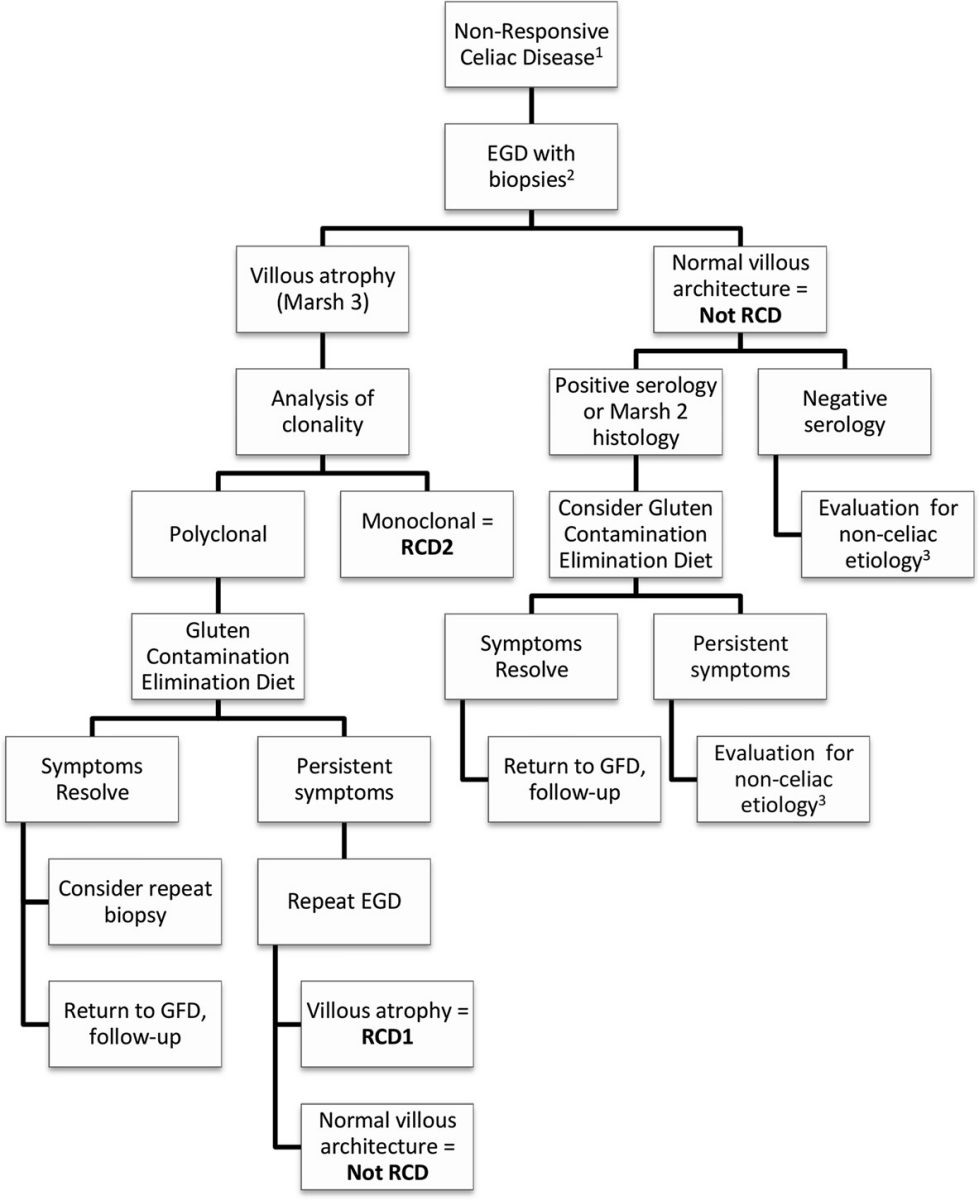
**Proposed diagnostic algorithm for non-responsive celiac disease.** 1. Dietary compliance should be assessed by an experienced dietitian to rule-out continued gluten exposure. 2. Consider colonoscopy with biopsies if symptoms clinically warrant. 3. To include testing for pancreatic insufficiency, small bowel bacterial overgrowth, lactose intolerance (based on clinical symptoms). EGD, esophagogastroduodenoscopy; RCD, refractory celiac disease; RCD1, refractory celiac disease type 1; RCD, refractory celiac disease type 2; GFD, gluten-free diet.

There are a number of recent articles discussing the evaluation of NRCD, with a focus on the approach to finding other underlying etiologies, such as inadvertent gluten ingestion or microscopic colitis, that may explain persistent symptoms [6,26,27]. It was not the intention of this study to duplicate this topic; rather we aimed to demonstrate that a number of GFD-adherent NRCD patients who are classified as RCD1 may not actually be refractory to dietary therapy. We propose a diagnostic algorithm (Figure 1) that would avoid misclassification of non-refractory patients, prevent unwarranted immunomodulatory therapy and preclude further unnecessary and often costly testing and imaging modalities employed in the search for non-CD etiologies.

Most of the patients referred to our celiac center for persistent symptoms, elevated serology, or enteropathy have unintentional dietary gluten secondary to a lack of proper dietary education. Therefore, our initial investigation begins with a comprehensive interview and evaluation by an experienced dietitian; only those patients *without* an apparent source of continued gluten exposure undergo further medical evaluation and consideration for the GCED.

After ensuring dietary compliance, the primary focus of an NRCD evaluation should be to rule-out the presence of RCD2, as this tends to follow an aggressive clinical course, with a high mortality rate primarily due to the development of intestinal lymphoma. Early recognition of RCD2 allows timely and more aggressive therapy, and encourages the use of diagnostic modalities such as video capsule endoscopy and CT enterography in order to exclude malignancy [15]. We recommend repeat EGD with biopsies as the first investigation in GFD-adherent NRCD patients to differentiate those with celiac enteropathy, and therefore possibly RCD, from those without persistent mucosal disease. Patients with villous atrophy (i.e. Marsh 3 histology) should undergo immunophenotyping of the intraepithelial lymphocyte population by CD3/CD8 immunostaining or T-cell receptor clonal rearrangement analysis by PCR. Whereas a monoclonal population equates to RCD2, the presence of a polyclonal IEL population should not equate to RCD1 without first a trial of the GCED. Only those patients who do *not* symptomatically and histologically respond to the diet should be diagnosed with RCD1 and treated appropriately [16,28].

Persistent positive tTG IgA serology cannot be used to differentiate RCD from those without sustained mucosal damage, nor can it be used to differentiate RCD subtypes [16]. Furthermore, studies have shown that normalization of tTG serology does not necessarily correlate with healed mucosa [29,30]. These reasons highlight the importance of endoscopy in the evaluation of NRCD patients. Nonetheless, tTG is a useful marker for dietary gluten contamination [31-33]. Our highest response rate was seen in that subset of patients with high celiac serology, with only 1 patient not responding to the GCED and, ultimately, being diagnosed with RCD1. The significance of a weak positive serology is less clear, and other studies of the NRCD population have also found little correlation between low titer positivity and underlying etiology [26]. In our study, there was no difference between those patients who transitioned from high to weak positive serology and those patients who went from high to negative serology.

As Marsh 2 histology has, by definition, normal villi, we do not include this classification among those patients with potential RCD, despite the persistence of pathologic features. However, in those symptomatic CD patients with elevated serology, neither do we consider Marsh 2 lesions to be indicative of disease remission. 2 patients in this study had Marsh 2 histology prior to the GCED; both improved serologically and symptomatically and one underwent repeat biopsy with subsequent normal (Marsh 0) histology. For this reason we recommend that those patients with Marsh 2 histology undergo the GCED prior to any further work-up/evaluation. Our study also included two symptomatic patients with positive celiac serology yet Marsh 1 histology prior to initiation of the GCED; both of whom had subsequent resolution of symptoms and improved serology. Marsh 1 lesions would be consistent with disease remission, suggesting that these patients’ diarrheal symptoms were secondary to irritable bowel syndrome (IBS) or an underlying etiology other than gluten contamination. However, while we recognize that any diet modification carries with it some degree of placebo effect, the correlation of symptom improvement with a change in celiac serology is intriguing and the fact that both patients were subsequently able to return to their traditional GFD further argues for a non-placebo response. It should also be noted that one patient’s endoscopic report documented visualization of signs of villous atrophy. This raises the possibility of patchy villous recovery and subsequent missed villous lesions by biopsy [34,35]. Given this response, we believe that a trial of the GCED should be considered in a symptomatic NRCD patient with positive celiac serology, despite apparently healthy villi.

Eighty-eight percent of our patients in this retrospective study were female. Although, as with other autoimmune diseases, there is a female predominance in CD, we were surprised that only 2 men were treated with the GCED since its development in 2005. As this study evaluated only those patients with NRCD who were treated with the GCED, we should not assume that this represents the entirety of our GFD-diet adherent NRCD population. However a previous study, after discounting those patients with inadvertent gluten ingestion, had an 81% female predominance in their NRCD population, supporting just such an assumption [27]. Likewise, a study evaluating duodenal recovery in CD patients found that male gender was independently associated with achieving Marsh 0-1 histology, with females comprising 77% of the study population but 85% of their nonresponsive category [29].

Owing to the retrospective study design, a major limitation of our study is the absence of repeat biopsies in some of our study patients, with 5 patients not undergoing endoscopy prior to the GCED and only 5 patients being biopsied after completing the GCED. Whereas the definition of RCD mandates persistent symptoms and, thus, an asymptomatic patient would no longer meet criteria for RCD, documentation of mucosal healing in those patients who responded clinically and serologically would strengthen the study results. Moreover, a disease-specific celiac symptom index would have aided in reliably assessing symptom resolution. Although compliance with the GCED was assessed by dietitian interview, we must consider the reality that strictly adhering to such a limited diet is difficult and likely more costly than a standard strict GFD given the focus on fresh, non-processed foods; these factors may affect adherence disclosure. Our study is also limited by lack of information on NRCD patients that were not started on the GCED, and we must recognize the possibility of a selection bias toward expected response to the GCED among our study subjects. Additionally, 8 patients who met NRCD criteria and were instructed on the GCED by our nutritionist never followed-up with our clinic and were therefore not included in our study. As it is our dietitian’s experience that GCED patients typically contact her periodically for clarification on diet, these patients likely did not start the diet. However, some of these patients may have complied with the diet and simply followed-up with their primary GI physician; as such, we may be missing useful data. Lastly, as it was our decision to include all NRCD patients who initiated the GCED, symptomatic patients with both negative serology and healed mucosa were not excluded; 75% of the noncompliant patients met this description, as well as one of the compliant patients. These patients essentially had little evidence to suggest that removal of trace gluten contamination would significantly affect their symptoms.

## Conclusions

The GCED may be an effective therapeutic option for NRCD patients that have already failed a well-documented strict GFD and may aid in differentiating those patients reacting to trace amounts of gluten contamination from those who truly have RCD1. By avoiding an inaccurate diagnosis of RCD1, patients are able to avoid corticosteroids or immunotherapy. The expense and potential adverse health effects of this type of therapy make a dietary solution, aimed at the underlying etiology, particularly attractive. Moreover, a successful trial of the GCED in those patients with a high likelihood of having gluten cross-contamination, for instance those with high celiac serology, would avoid further unnecessary medical work-up. Most patients who respond to a 3-6 month course of the GCED are subsequently able to return to a traditional GFD without return of symptoms. Multi-center studies implementing the GCED in subjects affected by NRCD despite strict diet-adherence should be performed to confirm our data and, if confirmed, to re-evaluate the actual incidence of RCD1 as it may currently be over-estimated.

## Abbreviations

CD: Celiac disease; RCD: Refractory celiac disease; NRCD: Non-responsive celiac disease; GFD: Gluten-free diet; GCED: Gluten contamination elimination diet.

## Competing interests

The authors have no competing interests to declare.

## Authors’ contributions

The authors’ responsibilities were as follows—JH: designed the study, conducted the research, and was the primary author for the manuscript; PC: contributed to the conception and design of the study and created the subject database; EL, MM and AF (Principal Investigator): contributed to the conception and design of the study, interpretation of results, and assisted in writing the manuscript. All authors approved the final version of the manuscript.

## Pre-publication history

The pre-publication history for this paper can be accessed here:

http://www.biomedcentral.com/1471-230X/13/40/prepub
